# Neuronal α‐amylase is important for neuronal activity and glycogenolysis and reduces in presence of amyloid beta pathology

**DOI:** 10.1111/acel.13433

**Published:** 2021-07-14

**Authors:** Elin Byman, Isak Martinsson, Henriette Haukedal, The Netherlands Brain Bank, Gunnar Gouras, Kristine K. Freude, Malin Wennström

**Affiliations:** ^1^ Clinical Memory Research Unit Department of Clinical Sciences Malmö Lund University Malmö Sweden; ^2^ Experimental Dementia Research Unit Department of Experimental Medical Science BMC B11 Lund University Lund Sweden; ^3^ Department of Veterinary and Animal Sciences Faculty of Health and Medical Sciences University of Copenhagen Frederiksberg Denmark; ^4^ Netherlands Institute for Neuroscience Amsterdam The Netherlands

**Keywords:** alpha‐amylases, Alzheimer's disease, amyloid beta‐peptides, calcium imaging, glycogen, induced pluripotent stem cells, tendamistat

## Abstract

Recent studies indicate a crucial role for neuronal glycogen storage and degradation in memory formation. We have previously identified alpha‐amylase (α‐amylase), a glycogen degradation enzyme, located within synaptic‐like structures in CA1 pyramidal neurons and shown that individuals with a high copy number variation of α‐amylase perform better on the episodic memory test. We reported that neuronal α‐amylase was absent in patients with Alzheimer's disease (AD) and that this loss corresponded to increased AD pathology. In the current study, we verified these findings in a larger patient cohort and determined a similar reduction in α‐amylase immunoreactivity in the molecular layer of hippocampus in AD patients. Next, we demonstrated reduced α‐amylase concentrations in oligomer amyloid beta 42 (Aβ_42_) stimulated SH‐SY5Y cells and neurons derived from human‐induced pluripotent stem cells (hiPSC) with PSEN1 mutation. Reduction of α‐amylase production and activity, induced by siRNA and α‐amylase inhibitor Tendamistat, respectively, was further shown to enhance glycogen load in SH‐SY5Y cells. Both oligomer Aβ_42_ stimulated SH‐SY5Y cells and hiPSC neurons with PSEN1 mutation showed, however, reduced load of glycogen. Finally, we demonstrate the presence of α‐amylase within synapses of isolated primary neurons and show that inhibition of α‐amylase activity with Tendamistat alters neuronal activity measured by calcium imaging. In view of these findings, we hypothesize that α‐amylase has a glycogen degrading function within synapses, potentially important in memory formation. Hence, a loss of α‐amylase, which can be induced by Aβ pathology, may in part underlie the disrupted memory formation seen in AD patients.

## Graphical abstract

Alpha‐amylase and glycogen are found in neuronal synapses. Inhibition of alpha‐amylase activity with Tendamistat increases neuronal glycogen load and causes calcium dyshomeostasis. Presence of amyloid beta is associated with reduced alpha‐amylase and glycogen load.

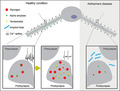

## 1 INTRODUCTION

Alzheimer's disease (AD) is the most common form of dementia and is neuropathologically characterized by the presence of amyloid beta (Aβ) plaques and neurofibrillary tau‐tangles (NFT) (Bloom, 2014). The pathological changes are thought to begin several years before symptoms appear (Jack et al., 2010; Sperling et al., 2011), and one of the earliest changes, besides the accumulation of Aβ and NFT, is reduced brain glucose metabolism (Mosconi, 2005). Since glucose constitutes the main energy source of the brain, impaired glucose metabolism has been suggested to be one of the major events underlying the neuronal loss and synaptic dysfunction seen in AD patients (Harris et al., 2012). Brain glucose metabolism is dependent on a constant uptake of glucose from the periphery, but as a backup mechanism, brain cells store and use glycogen, a multibranched polysaccharide produced by glycogen synthase. The production of glycogen occurs foremost in astrocytes (Ibrahim et al., 1975) and is degraded when energy is needed for the cell itself or, as studies suggest, is further converted into lactate, which is shuttled into neurons where it is used for energy (Bastian et al., 2019), memory formation (Duran et al., 2013; Gibbs, 2015), and neurotransmitter production (Hertz et al., 2015). This hypothesis has in recent years been challenged (Dienel, 2017; Drulis‐Fajdasz et al., 2018; Lundgaard et al., 2015) and several studies have shown that neurons can produce and degrade glycogen on their own (Duran et al., 2019; Saez et al., 2014; Sinadinos et al., 2014). This glycogenolysis appears to be crucial for neuronal function, as studies have shown that mice lacking neuronal glycogen synthase have impaired memory and long‐term potentiation (LTP) (Duran et al., 2019). The role of glycogen in memory formation, together with the known AD associated reduction of glucose metabolism, makes it tempting to speculate that impairment in glycogenolysis could in part underlie the memory decline and cognitive impairment seen in AD.

Degradation of brain glycogen is known to be executed by two enzymes called glycogen debranching enzyme and glycogen‐phosphorylase. However, we have recently demonstrated the presence of an additional glycogen degrading enzyme in the brain (Byman et al., 2018). This enzyme, called alpha (α)‐amylase is foremost known to be expressed in the salivary gland (Chopra & Xue‐Hu, 1993) and pancreas (Whitcomb & Lowe, 2007), but it can also be found in the intestines (Date et al., 2020), liver (Koyama et al., 2001), muscle, lung, and several other organs (Whitten et al., 1988). By staining a small subset of human postmortem hippocampal tissue, we showed that α‐amylase can be found in astrocytes (Byman et al., 2019), pericytes, and neurons (Byman et al., 2018). The neuronal α‐amylase was specifically located in structures resembling dendritic spines, which was clearly visible in non‐demented individuals (NC), but much less or completely lost in AD patients (Byman et al., 2018). The impact of AD pathology on brain α‐amylase was further verified by qPCR gene expression analysis of brain tissue, which showed that α‐amylase gene expression was significantly lower in AD brains compared to NC and correlated negatively with amyloid beta and NFT load (ABC and Braak staging). Whether the neuronal α‐amylase plays a role in glycogen degradation and whether a loss of the enzyme could lead to failure of glycogen dependent function such as LTP remains to be investigated, but we have shown that individuals with high copy numbers (CN) (>10) of the salivary α‐amylase isoform perform significantly better on an episodic memory test and also show significantly lower hazard ratio for developing AD (Byman et al., 2020). Since we also found a correlation between α‐amylase CN and brain α‐amylase production, we hypothesized that individuals with high α‐amylase CN may be protected against AD and have better cognitive abilities due to a higher amount of glycogen degrading α‐amylase in synapses. This challenging hypothesis needs to be tested, where the first step is to determine the cellular localization and function of neuronal α‐amylase. In the current study, we use human AD and NC postmortem hippocampal tissue and three different neuronal cell models; primary mouse neurons, hiPSC‐derived neurons with and without presenilin 1 (PSEN1) mutations and SH‐SY5Y cells to investigate whether (a) our previous observed reduction of α‐amylase in dendritic spine‐like structures in AD patients can be verified in a larger cohort; (b) Aβ has a direct impact on neuronal α‐amylase; (c) neuronal α‐amylase plays a role in glycogenolysis; (d) α‐amylase is localized in synaptic spines and associated with glycogen granules; and finally, (e) inhibition of α‐amylase activity affects neuronal activity using calcium imaging.

## 2 RESULTS

### 2.1 Lower alpha‐amylase immunoreactivity in neuronal processes of AD patients

**FIGURE 1:**
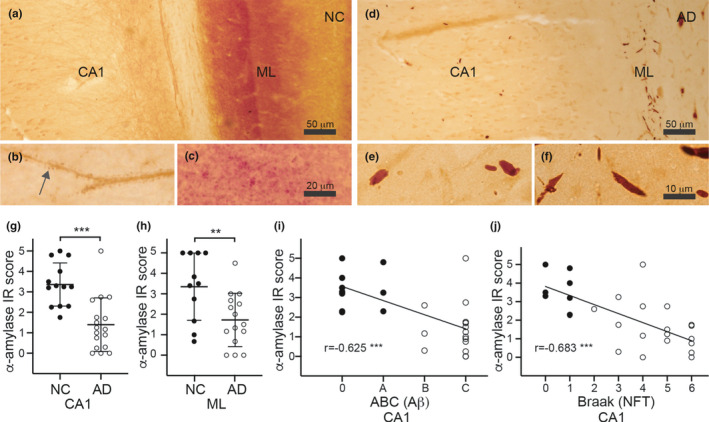
Immunohistochemical staining of alpha (α)‐amylase of human hippocampal postmortem tissue. Image in (a) shows an α‐amylase staining of Cornius ammonis 1 (CA1) and the molecular layer (ML) in a non‐demented control (NC). Higher magnification of the CA1 region in (b) shows that the α‐amylase immunoreactivity (IR) is found in dendritic spine‐like structures (DS). Higher magnification of the ML region in (c) shows that α‐amylase IR in ML forms a grained pattern. Image in (d) represent an α‐amylase staining of CA1 and ML in a patient with Alzheimer's disease (AD). Higher magnification of the CA1 region in (e) shows α‐amylase IR in form of Hirano bodies, which is also seen in the ML region in (f). Graph in (g and h) shows that scores of the DS α‐amylase IR in CA1 (g) and scores of grained α‐amylase IR in ML (h) are significantly higher in NC compared to AD. Scatterplot in (i–j) demonstrates a negative correlation between DS α‐amylase IR in CA1 and amyloid beta (Aβ) ABC scores (i) and neurofibrillary tangles (NFT) Braak scores in (j). Scale bar in (a and d) = 50 μm, in b–c = 20 μm and (e–f) = 10 μm. Graphs in (g–h) is presented as mean ± SD, and statistical analysis was done using *t* test. The correlation analyses in (i–j) were done using Spearman correlation test. ***indicates *p*‐value < 0.001, **indicates *p*‐value < 0.01

We initiated our study by scoring α‐amylase staining intensity in CA1 and ML of hippocampus in non‐demented controls (NC) and AD cases. As reported earlier, the α‐amylase immunoreactivity in NC was detected in structures resembling dendritic spines (Figure 1a,b). Also, the ML of NC showed an intense α‐amylase staining, yielding a grained appearance (Figure 1a,c). The immunoreactivity in ML and CA1 was weaker in AD patients, and the presence of Hirano bodies (HB) was detected in both areas (Figure 1d‐f). The difference in α‐amylase immunoreactivity was confirmed by our semiquantitative scoring, which showed significantly lower scores of dendritic spines in CA1 and ML grains in AD patients compared to NC (Figure 1g,h). The scores of CA1 correlated with ABC and Braak scores (Figure 1i,j). Also scores of ML correlated with both ABC and Braak scores (*r* = −0.458, *p* = 0.029 and *r* = 0.428, *p* = 0.019, respectively). The mean age of NC did not significantly differ compared to mean age of AD patients (76 ± 13 vs 80 ± 11, *p* = 0.415), and the gender distribution was similar in the NC and AD groups (54% and 59% females, respectively). Furthermore, the difference in scores of both CA1 and ML between ND and AD as well as the correlation with ABC and Braak scores remained significant after analysis using age or gender as a co‐variate (data not shown).

### 2.2 High presence of amyloid beta is associated with reduced alpha‐amylase levels

**FIGURE 2:**
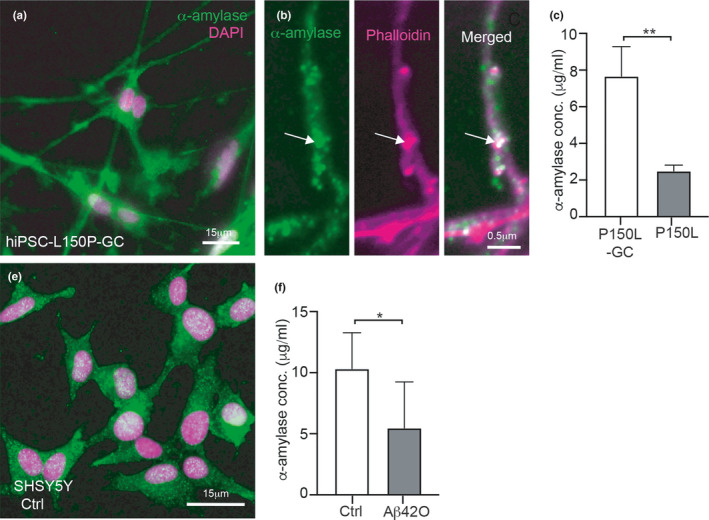
Alpha‐amylase in hiPSC‐derived neurons and oligomer amyloid beta‐42 stimulated SHSY5Y cells. Image in (a) shows hiPSC‐derived neurons without mutation in *PSEN1* gene (isogenic control) (L150P‐GC) immunoflourescently stained against salivary α‐amylase (green) with DAPI marker nuclei (magenta). A hiPSC neuronal process is shown in higher magnification in (b) where the arrow indicates α‐amylase (green) and phalloidin (magenta) in close association. Graph in (c) shows the significant lower levels of α‐amylase in L150P compared to L150P‐GC. Image in (e) shows control (ctrl) SH‐SY5Y cells immunofluorescent staining of α‐amylase (green), and nuclei are visualized with DAPI (magenta). The graph in (f) shows significantly lower α‐amylase concentrations in amyloid beta 42 oligomers Aβ42O stimulated cells compared to Ctrl. Scale bar in (a and e) indicates 15 μm, and scale bar in (b) indicates 0.5 μm. Statistical analysis was done with *t* test, and values in graphs (c and f) are presented as mean ± SD. ***indicates *p*‐value < 0.001, **indicates *p*‐value < 0.0, *p‐value < 0.05

To investigate whether the loss of neuronal α‐amylase that we observed in AD cases is linked to Aβ pathology, we analyzed α‐amylase levels in neurons derived from hiPSC cells containing a familial AD mutation in the *PSEN1* gene (L150P) and its isogenic control (L150P‐GC). Immunostaining of the cells showed that α‐amylase was found in processes, nuclei and cytosols of hIPSC cells (Figure 2a) and that the α‐amylase in processes were closely associated with phalloidin‐positive protrusions (Figure 2b). Further analysis showed that levels of α‐amylase were significantly lower in L150P cells compared to L150P‐GC (*p* = 0.003) (Figure 2c). To also investigate the direct effect of Aβ_42_ on α‐amylase, we next stimulated SH‐SY5Y with either vehicle control or oligomer Aβ_42_. The SH‐SY5Y cells showed α‐amylase immunoreactivity in the whole cell (Figure 2e), but the concentration of the enzyme was lower in cells stimulated with Aβ_42_O compared to vehicle control (*p* = 0.032) (Figure 2f). No significant difference in cell death measured by LDH cytotoxicity assay between the two stimulations was found (0.16 (015–0.19) vs 0.14 (0.12–0.18) *p* = 0.22).

### 2.3 Reduction of alpha‐amylase levels and activity is associated with higher glycogen storage

To understand the significance of a reduction of α‐amylase, we silenced the gene expression of α‐amylase in SH‐SY5Y using siRNA technique and investigated thereafter changes in glycogen load.

**FIGURE 3:**
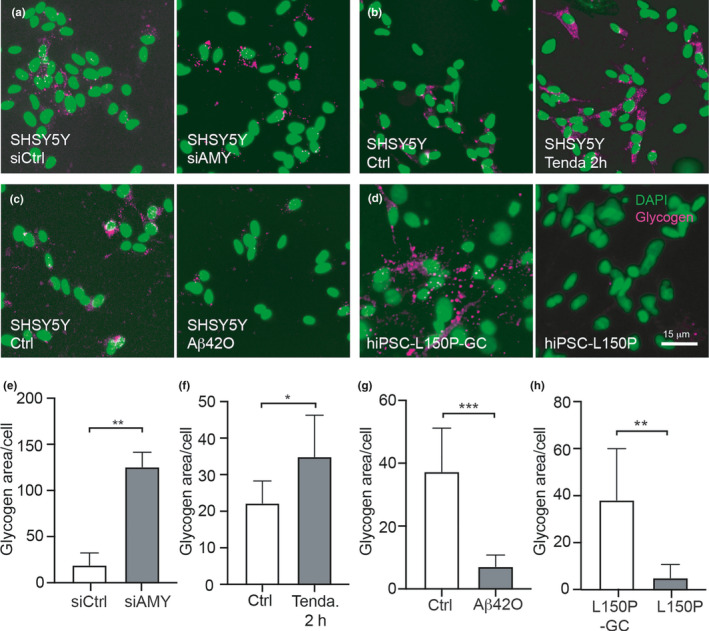
Glycogen in siRNA transfected SH‐SY5Y cells, Tendamistat or amyloid beta‐42 stimulated SH‐SY5Y cells and hiPSC‐derived neurons. (a–c) show representative images of SHSY5Y cells immunostained against glycogen (magenta) and cell nuclei marker DAPI (green) after being transfected with α‐amylase siRNA (siAMY) and non‐targeting siRNA (siCtrl) (a), stimulated with Tendamistat for 2 h (Tenda 2 h) and vehicle control (Ctrl) (b), and stimulated with Amyloid beta 42 oligomers (Aβ42O) and vehicle control (Ctrl) (c). Image in (d) shows glycogen (magenta) and DAPI (green) in neurons derived from hiPSC with mutation in PSEN1 (L150P) and isogenic control (L150P‐GC). Scale bar indicates 15 μm. Graph in (e) shows significant larger glycogen area/cell in SH‐SY5Y cells transfected with siAMY compared to siCtrl). Graph in (b) demonstrates significantly larger glycogen area/cell in SH‐SY5Y cells stimulated with Tenda. 2 h compared compared to Ctrl. Graph in (c) shows significantly smaller glycogen area/cell in Aβ42O stimulated SH‐SY5Y cells compared to Ctrl. Graph in (d) demonstrates significantly smaller glycogen area /cell in L150P compared L150P‐GC. Statistical analysis was done using *t* test, and data are presented as mean ± SD, ***indicates *p*‐value < 0.001, **indicated *p*‐value < 0.01, and *indicates *p*‐value < 0.05

The cells transfected with α‐amylase siRNA (siAMY) showed significantly reduced α‐amylase relative gene expression (about 45%) and α‐amylase protein concentration (about 50%) compared to non‐targeting siRNAs (siCtrl) (0.0013 ± 0.0006 vs 0.0024 ± 0.0012, *p* = 0.0079 and 2.5 ± 0.80 vs 5.13 ± 1.19, *p* = 0.049, respectively). Glycogen analysis using ImageJ showed that siAMY transfected cells had significantly higher glycogen area/cell compared to siCtrl (*p* = 0.0021) (Figure 3a). Cell death measurement with LDH cytotoxicity assay showed no significant difference between siCtrl and siAMY (0.66 ± 0.038 vs 0.65 ± 0.045, *p* = 0.87). To verify our findings, we also investigate glycogen load in SH‐SY5Y after stimulation with α‐amylase inhibitor Tendamistat, a polypeptide that strongly binds to α‐amylase and inhibits its hydrolytic activity (Vertesy et al., 1984). Stimulation with Tendamistat for 2 h led to a significantly higher glycogen area/cells compared to control (*p* = 0.036) (Figure 3b). The glycogen area/cell was still higher after 24 h of Tendamistat stimulation compared to control, but the difference did not reach significance (10.1 ± 6.5 vs 17.1 ± 7.7 *p* = 0.14). Cell death measurement with LDH cytotoxicity assay showed no significant difference between control and 2 h of Tendamistat stimulation (0.12 ± 0.018 vs 0.13 ± 0.017) or between control and 24 h of Tendamistat stimulation (0.15 ± 0,013 vs 0.016 ± 0,013, *p* = 0.09) *p* = 0.14).

### 2.4 High presence of amyloid beta is associated with reduced glycogen storage

Since both L150P hiPSC and Aβ_42_ stimulated SH‐SY5Y cells showed lowered α‐amylase levels, we investigated whether glycogen load in these cells was increased. To our surprise, our analysis instead showed significantly lower glycogen area/cell in Aβ_42_O stimulated SH‐SY5Y compared to vehicle control (*p* = 0.0002) (Figure 3c and g). A similar effect was seen in hiPSC with L150P mutation compared to isogenic control (*p* = 0.0049) (Figure 3d and h).

**FIGURE 4:**
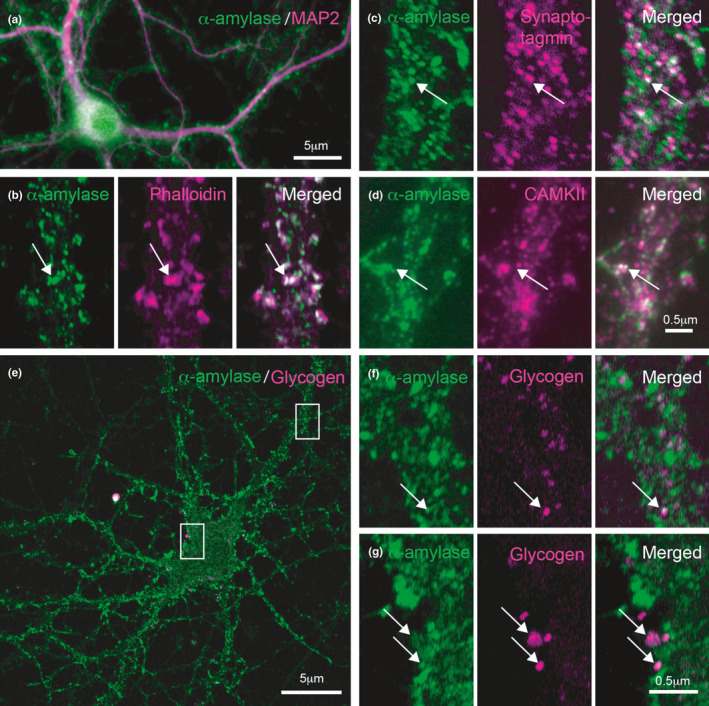
Cellular localization of alpha (α)‐amylase in primary mouse neurons. Image in (a) shows immunofluorescent staining of α‐amylase with antibody directed against salivary α‐amylase (green) and neuronal marker microtubule‐associated protein 2 (MAP2) (magenta). The α‐amylase immunoreactivity is seen in the cell body (arrowhead) and along the dendrites (arrow). Confocal image in (b) shows a primary mouse neuronal process where α‐amylase staining (green) and phalloidin (magenta) are in close association. Confocal image in (c) shows immunofluorescent staining of primary mouse neuronal process where α‐amylase (green) and synaptotagmin (magenta) are in close association (arrow). Confocal image in (d) shows a close association between α‐amylase (green) and CAMKII (magenta) (arrow). Confocal image in (e) shows a primary mouse neuron stained against α‐amylase (green) and glycogen (magenta). The white squares indicate magnification of the area seen in (f and g). Arrows in (f‐g) indicate an close association between α‐amylase and glycogen. Scale bar in (a and e) indicates 5 μm, scale bar in (d and g) indicates 0.5 μm

### 2.5 Alpha‐amylase co‐localize with synaptic markers

To further verify the presence α‐amylase in neurons, and determine its subcellular localization, we immunostained isolated primary mouse neurons against different neuronal markers together with αamylase. The staining showed α‐amylase immunoreactivity in the nucleus, cytosol, and neuronal processes of MAP2‐positive neurons (Figure 4a). The α‐amylase staining associated with the processes appeared dotted and were found scattered along and enclosing the MAP2‐positive processes (Figure 4a). This staining pattern resembled the neuronal α‐amylase staining we found in hippocampi of NC and similar staining pattern of α‐amylase could be seen when the primary neurons were stained with two other α‐amylase antibodies (S1). The dotted α‐amylase along the neuronal processes were closely associated with synaptic protrusions stained with the actin dye phalloidoin (Figure 4b), presynaptic marker synaptotagmin‐1 (Figure 4c), and synaptic marker CAMKII (Figure 4d). A co‐staining with glycogen further revealed a close association between α‐amylase and some glycogen granules within the cytosol and along the dendrites (Figure 4e‐k).

### 2.6 Inhibition of alpha‐amylase alters spontaneous calcium oscillations in neurons

**FIGURE 5:**
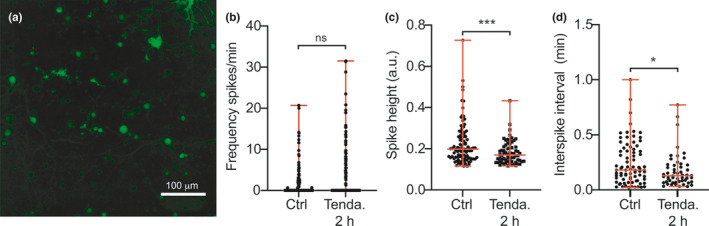
Calcium imaging of Tendamistat treated primary mouse neurons. Micrograph in (a) shows maximum intensity projections of the imaging time series of primary neuron culture labeled with Fluo4 AM. Scale bar in a indicates 100 μm. Graph in (b) shows frequency of spikes per minutes in primary mouse neurons stimulated with α‐amylase inhibitor Tendamistat for 2 h (Tenda. 2 h). Graph in (c) demonstrates significantly lower amplitude of calcium concentrations (spike height) after 2 h stimulation with Tendamistat compared to vehicle control (Ctrl). Graph in (c) demonstrates significantly shorter inter‐spike intervals after 2 h Tendamistat stimulation compared to Ctrl. Statistical analysis was performed by Mann–Whitney *U* test, and data are presented as median and range ***indicates *p*‐value < 0.001 and *indicates *p*‐value < 0.05

As our results indicate α‐amylase is implicated in glycogen degradation and since it appears to be localized proximal to synapses, it is of interest to investigate whether the enzyme is needed for neuronal communication. We therefore analyzed the calcium transients (Figure 5a) in primary mouse neurons after inhibiting α‐amylase activity with Tendamistat. While we did not see a statistically significant change in spike frequency (*p* = 0.248) (Figure 5b), Tendamistat significantly lowered the amplitude of intracellular calcium concentrations (Figure 5c) and led to shorter inter‐spike intervals (Figure 5d) after 2 h of Tendamistat stimulation compared to non‐stimulated controls (*p* = 0.001 and *p* = 0.04, respectively).

## 3 DISCUSSION

Our study showed that α‐amylase IR scores are significantly decreased in CA1 and ML of AD patients compared to NC and that the scores correlate negatively with both ABC (Aβ) and Braak (NFT) scores. By staining primary mouse neurons, we also showed that α‐amylase is present in dendritic spines and the cell body, where it co‐localizes with glycogen. The α‐amylase production appears to be affected by Aβ_42,_ as we found significantly reduced α‐amylase levels in both hiPSC neurons containing the PSEN1 gene mutation and SH‐SY5Y cells stimulated with oligomer Aβ_42_. A reduction of α‐amylase activity, either by silencing the gene expression with siRNA or by inhibiting the activity with Tendamistat, led to an increase in glycogen area/cell in SH‐SY5Y cells, but both hiPSC with PSEN1 mutation and oligomer Aβ_42_ stimulated SH‐SY5Y cells showed lowered glycogen area/cell compared to control. Finally, inhibition of α‐amylase activity altered neuronal calcium oscillations, specifically lowering amplitudes and shortening inter‐spike intervals.

In line with our previous observation (Byman et al., 2018), staining for α‐amylase yielded a staining pattern resembling dendritic spines in CA1, and the immunoreactivity of these structures in this region was lowered in AD patients. The α‐amylase staining also showed a previous not reported intense α‐amylase staining in ML of non‐demented controls, which reduced in strength in AD patients. The grained pattern could reflect the high density of dendrites in ML, which extends from the granular cells of the dentate gyrus (Amaral et al., 2007). These granular cells and the glutamatergic pyramidal neurons located in CA1 are known to be important for learning and memory (Lopez‐Rojas & Kreutz, 2016), which raises the question whether α‐amylase has a function within synapses of these cells related to learning and memory formation. To further verify that α‐amylase is truly found in synapses and since it is difficult to clearly visualize synapses in tissue, we analyzed α‐amylase in isolated primary mouse neurons, a neuronal model with functional synapses. Our results showed that α‐amylase is found associated with dendritic protrusions positive for the F‐actin marker phalloidin as well as the synaptic markers synaptogamin‐1 and CAMKII. The former synaptic marker is commonly known as a regulator of the SNARE complex in pre‐synapses but is also found in CA1 post‐synapses where it regulates LTP‐dependent exocytosis of AMPA receptor (Wu et al., 2017). CAMKII, which is foremost found in post‐synapses, is also known to be implicated in LTP and memory formation (Byth, 2014; Sacchetto et al., 2007). Of note, the imaging techniques used in this study are not sufficient enough to specify whether α‐amylase interacts with these synaptic markers or whether α‐amylase is specifically localized in pre‐ or post‐synapses or found in both compartments.

The reduction in α‐amylase IR found in our postmortem study could be due to the severe loss of neuronal synapses and dendritic spines seen in AD patients (Pereira et al., 2021). But since the α‐amylase immunoreactivity correlated negatively with Aβ ABC and NFT Braak scores, and since we found significantly lower concentration of α‐amylase in hiPSC‐derived neurons with PSEN1 mutation (which leads to increase Aβ_42_/Aβ_40_ ratio (Kumar‐Singh et al., 2006)), it may well be that Aβ has a direct negative impact on α‐amylase expression. The lowered α‐amylase concentration found in SH‐SY5Y cells after stimulation with Aβ_42_ oligomers support this idea.

Our finding of close association between α‐amylase and glycogen granules in the cell body and processes of primary mouse neurons are interesting given that the main function of α‐amylase throughout the body is to degrade polysaccharides (Singh & Guruprasad, 2014). It is thus not unlikely that α‐amylase has a similar role in neurons, degrading glycogen in order to free glucose needed for processes in the different neuronal compartments. Indeed, when we silenced α‐amylase in SH‐SY5Y cells using siRNA, we noted a significant increase in glycogen compared to control‐treated cells. Similar findings where seen when SH‐SY5Y cells were incubated with Tendamistat, a polypeptide that tightly binds to α‐amylase and inhibits its enzymatic activity (Vertesy et al., 1984). In view of these findings, we speculate that α‐amylase has a role in neuronal glycogenolysis, but whether this role is direct (i.e., degrades glycogen) or indirect (i.e., affects enzymes, kinases, and proteins implicated in glycogenolysis) remains to be investigated. Nevertheless, it is important to mention that although our studies point toward a glycogen degrading role for α‐amylase, we do not exclude the possibility that the enzyme has other, for us unknown, functions and has its impact on neurons by, for example, interaction with other proteins and ions.

To our surprise, even though oligomer Aβ_42_ stimulated SH‐SY5Y cells showed lowered α‐amylase concentration, glycogen load was equally lowered in these cells. Of note, patients with AD show both reduced glycogen load (Bass et al., 2015) and increased glycogen synthesis‐regulating GSK3beta (Reddy, 2013) which is shown to lower production of glycogen (Bass et al., 2015; Beurel et al., 2015). The Aβ‐induced reduction of both glycogen and α‐amylase in neurons suggests that there is a doubled impact on glycogen metabolism, that is, both a reduction in degradation and formation of glycogen. Such a scenario is of interest given the fact that neuronal glycogen is specifically important in LTP (Duran et al., 2019) and it is therefore tempting to speculate that the α‐amylase reduction in combination with lowered access to glycogen plays a role in the cognitive decline seen in AD patients. This idea would be in line with our previous study demonstrating lower hazard ratio for developing AD as well as significantly better results on episodic memory test in individuals with high AMY1A copy number variants (Byman et al., 2020). In view of the hypothesis, we expected our calcium imaging analysis to show an elimination of neuronal activity after inhibiting α‐amylase activity with Tendamistat. To our surprise, we instead found a non‐significant increase in frequency of calcium oscillations as well as lowered amplitude of intracellular calcium concentrations and inter‐spike intervals. It thus appears as if α‐amylase is not crucial for neuronal activity *per se*, but a lack of the enzyme induces an alteration which may be pathological. From this perspective, it is interesting that a recent study demonstrates lowered amplitude of intracellular calcium and higher frequency of calcium oscillations in motor cortex neurons of transgenic JNPL3 tauopathy mice (Wu et al., 2021). The calcium dyshomeostasis in these mice was linked to a selective downregulation of AMPA receptors, which regulates formation of LTP via postsynaptic abundance (Diering & Huganir, 2018). Whether a shortage of free glucose molecules affects AMPA receptor trafficking and calcium oscillation in neurons is not (to our knowledge) known, but oxygen / glucose deprivation (a model for ischemia) has been shown to both increase glutamate at synapses to excitotoxic levels (Sattler et al., 2000) and decrease AMPA receptors (Dennis et al., 2011). It is thus tempting to speculate that the calcium dyshomeostasis seen in the Tendamistat treated neurons is due to a shortage of glucose caused by altered glycogen degradation, which in the long run could affect AMPA‐dependent LTP formation.

Our work has some limitations that need to be addressed. The glycogen antibody (ESG1A9) used in our study has only been evaluated in few studies and given that there is still some debate whether healthy neurons produce and store glycogen, it is important to discuss the antibody's specificity. The ESG1A9 was produced in Professor Hitoshi Ashida laboratory and was evaluated using an ELISA with 15 different glycogen species as baits (6 from a natural sources (NSG), 5 enzymatically‐synthesized glycogens (ESG), starch, resistant glycogen (RG), and high molecular weight glycogen polymers). The analysis showed that the antibody binds to all NSG and ESG as well as to RS, but that complete digestion of glycogen into smaller fractions (29 kDa) by α‐amylase eliminate the binding of antibody (Nakamura‐Tsuruta et al., 2012). Staining using ESG1A9 has further been compared by periodic acid Schiff (PAS) staining (a staining commonly used for analysis of oligosaccharides/glycogen in tissue) in a study on glycogen load in mouse hippocampus. The two staining techniques showed similar pattern and the ESG1A9 staining was lost when the tissue was pretreated with α‐amylase (Oe et al., 2016). In view of these evaluation studies, we would like to argue that ESG1A9, if not completely specific, then at least binds with high affinity to glycogen. Another limitation concerns the specificity of Tendamistat. Although it is known that the small peptide (7,9 kDa) specifically binds and inhibits human α‐amylase activity, there are to our knowledge no studies investigating its impact on other glycogen degrading substances or other cellular processes. Hence, we cannot entirely exclude the possibility that the increase in glycogen load or calcium dyshomeostasis seen after Tendamistat is caused by an alternative pathway besides its impact on α‐amylase. Similarly, although the siRNA used in our study is generated to specifically interfere with α‐amylase mRNA translation, we cannot exclude the possibility that other genes may be effected as well. However, since both methods, based on two different principles, yield the same result we find it likely that α‐amylase does have glycogen degrading properties also in neurons. Finally, the results found in our study are primarily based on neuronal models, which is a limitation as cell cultures can never mimic the events happening in the brain. The studies on primary mouse neurons help us to clearly visualize the localization of α‐amylase as well as alterations in intracellular calcium as an indication of neuronal activity, but the model has the limitation of not being of human origin. The hiPSC‐derived neurons have few true synapses but have the advantage that it is a human neuronal model and mimics a clinical scenario. Finally, SH‐SY5Y cells are merely a neuroblastoma cell line, but it can, in contrast to hiPSC and primary neurons, be transfected by siRNA. Hence, each model has its own strengths and limitations, but we would like to argue that together they point toward the same direction, namely a role for α‐amylase in neuronal glycogen metabolism and communication.

## 4 CONCLUSION

To conclude, our study suggests that α‐amylase can be found in both human and mouse neuronal synapses and that loss of neuronal α‐amylase activity increases neuronal glycogen load and alters neuronal activity. Our study further shows that neuronal α‐amylase is reduced in AD patients and indicates that this loss may be due to a direct impact of Aβ_42_. Based on these results, we hypothesize that α‐amylase plays a role in glycogenolysis important for neuronal activity and that loss of α‐amylase in AD patients partly underlie the cognitive decline seen in these patients.

## 5 EXPERIMENTAL PROCEDURES

### 5.1 Individuals included in the study

Hippocampal samples from non‐demented controls (NC) (*n* = 13) (mean age 76 ± 13, 44% females) and clinically and postmortem verified patients with AD (*n* = 17) (mean age 80 ± 11, 49% females) (the Netherlands Brain Bank (NBB)) were fixed in paraformaldehyde (PFA), left in 30% sucrose for 3 days and sectioned into 40 mm sections. Autopsies were performed by the NBB at the designated premises of the VU Medical Center in Amsterdam (the Netherlands). Written informed consent for the use of brain tissue and clinical data for research purposes were obtained from all donors or their next of kin in accordance with the International Declaration of Helsinki and Europe´s Code of conduct for Brain Banking. The procedures and forms of the Netherlands Brain Bank were approved by the medical ethics committee of VU medical center in Amsterdam (the Netherlands), and the regional ethical review board in Lund has approved the study.

### 5.2 Immunohistochemical staining of brain tissue

Differences in α‐amylase immunoreactivity in ML and CA1 were analyzed by staining three hippocampal sections from each individual. The sections were quenched in 10% methanol and 10% H_2_O_2_ for 30 min in room temperature (RT) and then blocked in Impress reagent kit blocking solution (Vector Laboratories, Burlingame, RI) for 1 h in RT, followed by an incubation with rabbit anti‐AMY1A (Thermo Fisher Scientific, Waltham, MA) over night at 4℃. The following day sections were washed and incubated with Ig Impress reagent kit anti‐rabbit antibody (Vector Laboratories, Burlingame, RI) for 2 h at RT. The staining was developed by incubating the sections in 0.25 mg/ml diaminobenzidine and 0.012% H_2_O_2_ for 2 min. The α‐amylase immunohistochemical (IHC) staining intensity in CA1 (NC (*n* = 13) and AD (*n* = 17)) and ML (NC (*n* = 11) and AD (*n* = 15) (parts of dentate gyrus and ML of two NC and two AD patients were loss due to tissue fragility) of each section was semi‐quantitatively scored (0–5) by two independent blinded observers as no (0), very weak (1), weak (2), moderate (3), strong (4), and very strong (5) staining. The values were averaged and presented as mean α‐amylase IHC score.

### 5.3 Culturing SH‐SY5Y cells

Human neuroblastoma cell‐line SH‐SY5Y (Sigma‐Aldrich, St. Louis, MO, USA) was thawed and seeded out in Poly‐l‐lysine (Sigma‐Aldrich, St. Louis, MO, USA) coated 12‐well plates (Nunc, Thermo Fisher Scientific, Waltham, MA) and Lab‐Tek 8‐well chamber slides (Thermo Fisher Scientific, Waltham, MA) coated with either Poly‐l‐Lysine or collagen I rat tail (Thermo Fisher Scientific, Waltham, MA). The SH‐SY5Y cells were grown in medium containing 41.5% Minimum Essential Medium Eagle (Sigma‐Aldrich, St. Louis, MO, USA), 41.5% Nutrient mixture F‐12 Ham (Sigma ‐Aldrich, St. Louis, MO, USA), 15% Fetal bovine serum (Thermo Fisher Scientific, Waltham, MA), 1% l‐glutamine (Thermo Fisher Scientific, Waltham, MA), 1% MEM non‐essential Amino Acids (Sigma‐Aldrich, St. Louis, MO, USA) at 37℃, 5% CO_2_ and 5% H_2_O until 60%–80% confluency.

### 5.4 Differentiation and culturing of hiPSC

Human‐induced pluripotent stem cells (hiPSC) derived from one patient carrying the L150P PSEN1 mutation (Tubsuwan et al., 2016) and its gene‐corrected isogenic control (L150P PSEN1‐GC) (Poon et al., 2016) were thawed and cultured on Matrigel‐coated plates (TH Geyer, Renningen, Baden‐Württemberg) in Essential 8 (E8) media (Thermo Fisher Scientific, Waltham, MA) together with supplement. Every day the media were changed, and the cells were passaged every third day for about 2 weeks using 0.5 mM EDTA (B52, Thermo Fisher Scientific, Waltham, MA), before neural induction. Neural differentiation was performed according to a modified dual SMAD protocol (Shi et al., 2012), when hiPSC reached approximately 90% confluency. Briefly described, the media were changed into neural induction media (NIM) containing 50% DMEM/F12 (Sigma‐Aldrich, St. Louis, MO, USA), 50% Advanced neurobasal medium (Thermo Fisher Scientific, Waltham, MA), 1% N2 (Thermo Fisher Scientific, Waltham, MA), 1% B27 without retinoic acid (Thermo Fisher Scientific, Waltham, MA), 1% Glutamax (Thermo Fisher Scientific, Waltham, MA), 1% Non‐essential amino acid (NEAA, Thermo Fisher Scientific), 1% Pen/Strep (Sigma‐Aldrich, St. Louis, MO, USA), supplemented with the inhibitors 10 μM SMAD inhibitor (SMSgruppen, Rungsted, Denmark) and 0,1 μM Noggin analog (Sigma‐Aldrich, St. Louis, MO, USA). The cells were maintained in NIM (with change of media every day) for 12 days and thereafter uniform neuroepithelial sheets had appeared, and the neural progenitor cells (NPC) were passaged with Accutase (Thermo Fisher Scientific, Waltham, MA) into neural expansion media (NEM) containing growth factors 20 ng/ml FGF2 (ProSpec, Rehovot, Israel) and 20 ng/ml EGF (ProSpec, Rehovot, Israel) instead of the inhibitors. Following, NPCs were expanded with change of media every other day and thereafter seeded out in Poly‐l‐Ornithine (Sigma‐Aldrich)/laminin (Sigma‐Aldrich, St. Louis, MO, USA) with a density of 50,000 cells/cm^2^. By changing into neural maturation media (NMM), supplemented with 50 μM db‐cAMP (Sigma‐Aldrich, St. Louis, MO, USA), 200 μM Ascorbic acid (Sigma‐Aldrich St. Louis, MO, USA), 20 ng/ml BDNF (ProSpec, Rehovot, Israel) and 10 ng/ml GDNF (ProSpec, Rehovot, Israel), terminal neural differentiation was performed, and the cells matured for 7 weeks, with a partial media change every third day. The neurons were then fixed in 4% PFA for immunocytochemistry and protein lysates were harvested with M‐PER (Thermo Fisher Scientific, Waltham, MA) with cOmplete (Roche, Basel, Switzerland) and PhosSTOP (Roche, Basel, Switzerland).

### 5.5 Amyloid beta 42 oligomer stimulation

Oligomeric Aβ_42_ was prepared according to Brannstrom et al. (2014). Briefly, synthetic Aβ_42_ (AlexoTech, Umeå, Sweden) was dissolved in 20 mM NaOH; thereafter, 5 M NaCl was added, and the pH was neutralized with 200 mM phosphate buffer and finally diluted with ddH_2_O to a concentration of 100 mM and incubated on a shaker for 20 min at RT. Vehicle control was prepared by mixing 20 mM NaOH, 5 M NaCl, and 200 mM phosphate buffer in the same volumes as Aβ_42_ oligomers were dissolved in. SH‐SY5Y cells in 12‐well plates and 8‐well glass chambers grown to 80% confluency were thereafter stimulated with 1 μM Aβ_42_ oligomers (Aβ_42_O) in OptiMEM (Thermo Fisher Scientific, Waltham, MA) or with vehicle control in OptiMEM and incubated at 37℃, 5% CO_2_ and 5% H_2_O for 18 h. After incubation, the medium was collected and cells were lysed with mammalian cell lysis kit (Sigma‐Aldrich, St. Louis, MO, USA) containing phosphatase inhibitor cocktail 2 and 3 (Sigma‐Aldrich, St. Louis, MO, USA) or with Qiazol (Qiagen, Venlo, the Netherlands). Cells in 8 well glass chambers were fixed in 2% PFA.

### 5.6 SiRNA transfection

SH‐SY5Y cells grown in 12‐well plates and 8 well chambers until 60% confluency was subjected to small interfering RNA (siRNA) transfection according to manufacturer's instructions. In short, Lipofectamine RNAiMAX (Thermo Fisher Scientific, Waltham, MA) together with 40 nM ONTARGETplus non‐targeting, Human AMY2A SMARTpool siRNA (Horizon Discovery, Cambridge, UK) were diluted in OptiMEM (Thermo Fisher Scientific, Waltham, MA) and added to the cells. After 24 h, 50% of the medium containing Lipofectamine and siRNA:s was discarded and refilled with the same volume of freshly prepared siRNA and lipofectamine and incubated for additional 24 h. After incubation, the medium was collected and the transfected SH‐SY5Y cells in 12‐well plates were lysed with mammalian cell lysis kit (Sigma‐Aldrich, St. Louis, MO, USA) containing phosphatase inhibitor cocktail 2 and 3 (Sigma‐Aldrich, St. Louis, MO, USA). Cells in 8‐well glass chambers were fixed in 2% PFA. The experiment was repeated three times with two replicates for each condition.

### 5.7 Tendamistat stimulation

Polypeptide α‐amylase inhibitor Tendamistat (HOE‐467A from *Streptomyces tendea*, My BioSource, San Diego, CA) was dissolved in ddH_2_O to a concentration of 0.5 mg/ml. Tendamistat was then, prior to stimulation, further diluted in OptiMEM (Thermo Fisher Scientific, Waltham, MA) to a concentration of 1 μM and then added to SH‐SY5Y cells in 12‐well glass slides (Ibidi GmbH, Gräfelfing, Germany). Tendamistat stimulated cells and unstimulated cells (control) were incubated for 2 resp. 24 h in 37℃, 5% CO_2_ and 5% H_2_O. After incubation, the medium was collected, and cells were fixed in 2% PFA.

### 5.8 Lactate dehydrogenase cytotoxicity assay and Protein concentration measurement

Supernatant Medium from siRNA transfected, Aβ_42_O and Tendamistat stimulated SH‐SY5Y cells were centrifugated at 10,000 g for 10 min at 4℃ and the pellet discarded. Cytotoxicity detection assay (Roche, Basel, Switzerland), measuring released lactate dehydrogenase (LDH), was performed on medium supernatant according to manufacturer's instructions. Protein concentration measurement in cell lysates from siRNA transfected and Aβ_42_O stimulated SH‐SY5Y cells were done using PierceÔ BCA Protein assay kit (Thermo Fisher Scientific, Waltham, MA) according to manufacturer's instructions.

### 5.9 Alpha‐amylase ELISA

Protein concentration of α‐amylase in siRNA transfected and Aβ_42_O stimulated SH‐SY5Y cells as well as hiPSC were measured using an in‐house‐developed indirect ELISA. The capture antibody was evaluated by immunoprecipitation and Western blot (S2). Samples and Human salivary α‐amylase standard (Sigma‐Aldrich, St. Louis, MO, USA) were incubated at 4℃ overnight. The following day, plates were blocked for 1 h at RT with blocking solution (BS) (1% bovine serum albumin in phosphate buffer Saline with 0.25% tween) followed by incubation with rabbit polyclonal anti‐AMY1A (Thermofisher Scientific) diluted in BS for 2 h at RT on shake. Finally, goat‐anti‐rabbit horseradish peroxidase secondary antibody (Abcam, Cambridge, Great Britain) diluted in BS was added to the plate and incubated for 1 h at RT on shake. The plates were developed using TMB microwell peroxidase (KPL, Gaithersburg, USA); the reaction was stopped with 1 mM H_2_SO_4_ and measured at 450 nm using spectrophotometer Eon™ (Biotek, Winooski, VT). The intra‐ and inter‐assay variation coefficient (CV%) were 12.2% and 13.7%, respectively.

#### 5.9.1 Isolation of primary mouse neurons

Primary embryonic mouse neurons were prepared from the cortices and hippocampi of WT mouse embryos at embryonic day 15–17. Briefly, the pregnant mother was sacrificed by cervical dislocation after being put to sleep with isofluorane. The embryos were then removed and washed in 70% ethanol; following two rinses in Hank's basal salt solution with 0.45% glucose added, meninges were removed and the cortices and hippocampi dissected out. The neurons were dissociated by trypsinization and then triturated in Dulbecco's modified Eagle medium (DMEM) with 10% fetal bovine serum (FBS) and penicillin/streptomycin (Thermo Fisher, Waltham, MA). They were thereafter plated onto poly‐Dlysine coated culturing vessels and incubated in the DMEM media. After 3–5 h, the medium was exchanged for Neurobasal medium supplemented with B27 (Thermo Fisher, Waltham, MA), Lglutamine (Thermo Fischer, Waltham, MA), and penicillin/streptomycin (Thermo Fisher, Waltham, MA). The neurons were then maintained in Neurobasal media until 19–21 days in vitro, whereafter they were prepared for staining by fixation in 4% PFA for 15 min.

#### 5.9.2 Immunocytochemistry of primary mouse neurons, SH‐SY5Y cells and hiPSC‐neurons

Primary mouse neurons on glass slides were permeabilized by incubating the cells with 0.1% saponin (Sigma‐Aldrich, St. Louis, MO, USA) in 1% BSA/PBS^Ca2+,Mg2+^ and thereafter blocked with 1% BSA/PBS^ca2+,mg2+^ for 1 h at RT. Primary antibodies directed against; salivary α‐amylase (AMY1A aa 195‐223) (rabbit anti‐AMY1A Thermo Fisher Scientific, Waltham, MA), salivary α‐amylase (sheep anti‐salivary α‐amylase, Abcam, Cambridge, Great Britain), and pancreatic α‐amylase (AMY2B aa 74‐100, Thermo Fisher Scientific, Waltham, MA) were co‐incubated with primary antibodies against glycogen (mouse IgM‐anti‐glycogen, ESG1A9mAb; kind gift from professor Hitoshi Ashida at Kobe University), Microtubule‐associated protein 2 (MAP2) (mouse anti‐MAP2 Sigma‐Aldrich, St. Louis, MO, USA), synaptotagmin (guinea pig anti‐synaptotagmin 1, Synaptic systems, Göttingen, Germany), or CAM kinase II clone 6G9 (EDM Millipore, Burlington, MA, USA) diluted in 1% BSA/PBS^ca2+,mg2+^ for 2 h at RT. The cells were thereafter incubated with the appropriate secondary antibodies, that is, Alexa^562^ goat anti‐mouse IgM (Thermo Fischer Scientific, Waltham, MA), Alexa^488^ goat anti‐rabbit IgG, Alexa^350^ goat‐anti‐mouse (Thermo Fischer Scientific, Waltham, MA), and goat anti‐mouse Dylight 549 (Vector Laboratories, Burlingame, CA) or donkey anti‐sheep Biotinylated (Vector Laboratories, Burlingame, CB2A) or with Phalloidin TexasRed‐X probe (Thermo Fisher Scientific, Waltham, MA) 1 h at RT. Streptavidin‐Dylight 488 Conjugate (Vector Laboratories, Burlingame, CA) was added to cells incubated with Biotinylated donkey‐anti‐sheep antibody. Negative controls were performed in absence of antibodies against glycogen and AMY1A (S3) siRNA transfected, Aβ_42_O and Tendamistat stimulated SH‐SY5Y cells in 8‐ or 12‐well glass chambers as well as hiPSC cells on glass slides were immunostained against glycogen, MAP2, and salivary α‐amylase following the same procedure as described above. The slides were mounted with Vectashield Set mounting medium with or without DAPI (Vector Laboratories, Burlingame, CA).

Images of Primary mouse neurons were obtained with Confocal microscope (Zeiss LSM 800 with Airyscan) using 63X objective and Olympus AX70 microscope using 20× and 40× objective.

#### 5.9.3 Calcium imaging

Primary WT neurons seeded in Ibidi 8‐well slides were kept for 20 days in vitro. Prior to imaging, cells were treated with Tendamistat or vehicle control (ddH2O) for 2 h. 30 min before imaging 3 μM calcium indicator fluo‐4 AM was added to the neurons. Cells were imaged under a Nikon Eclipse Ti microscope at 10× with, 1.4 NA. Live cell imaging chamber (Okolab) was kept at 5% CO_2_ and 37℃. Cells were imaged every 100 ms for a duration of 2 min with an iXon Ultra CCD camera (ANDOR Technology). Time stacks were opened in FiJi, and individual regions of interest were marked around neuronal cell bodies (*n* = 108 Ctrl, Tendamistat *n* = 66). Fluorescence intensity over time was extracted, normalized, and processed with the MATLAB script PeakCaller (Artimovich et al., 2017).

Spike detection threshold was set at 10% over baseline. In order to process spike height and inter‐spike intervals, silent neurons were omitted as these interfere with amplitude and interval measurements. Data were then exported and analyzed in GraphPad Prism.

#### 5.9.4 Statistical analysis

Statistical analysis was performed using GraphPad Prism (version 8.1.2) and SPSS statistics (version 25). Analysis using Kolmogorov–Smirnov test showed that all values except LDH cytotoxicity values in Aβ_42_O stimulated SH‐SY5Y cells were normally distributed. Differences in α‐amylase IHC scores between NC and AD were analyzed using Student's *t* test. Correlations between α‐amylase IHC scores and ABC (Aβ) scores and Braak (NFT) scores were analyzed using Spearman correlation test. Differences in α‐amylase relative gene expression in siCtrl vs siRNA treated SH‐SY5Y were analyzed with ratio paired *t* test. Differences in α‐amylase concentration, LDH cytotoxicity and glycogen area/cell between siCtrl and siRNA treated SH‐SY5Y were analyzed with paired sample *t* test (three separate measurements with two replicates for each condition). Differences in α‐amylase concentration and glycogen area/cells in Aβ_42_O and Tendamistat stimulated SH‐SY5Y cells were analyzed by the use independent sample *t* test (6 technical replicates for each condition). Differences in LDH cytotoxicity assay in between control and Aβ_42_O stimulated SH‐SY5Y cells were analyzed with non‐parametric Mann–Whitney *U* test. Differences in α‐amylase concentration and glycogen area/cells between L150P and L150P‐GC cells were analyzed with independent sample *t* test (4 technical replicates for each condition). Differences in calcium frequency spikes, calcium amplitude, and interval spikes between unstimulated and Tendamistat stimulated primary mouse neurons were analyzed with non‐parametric Mann–Whitney *U* test. Values from paired and unpaired *t* tests are presented as mean ± SD and values from Mann–Whitney *U* test are presented as median and range. *p*‐values under 0.05 were considered significant.

## CONFLICT OF INTEREST

The authors declare that they have no competing interests.

## AUTHOR CONTRIBUTIONS

EB co‐designed the study, carried out the siRNA studies, RT‐qPCR, ELISA immunocytochemical stainings and analyzed the data. IM performed the calcium imaging and data analysis. HH generated the hiPSC. NBB collected the brain tissue samples and performed the neuropathological evaluation. GG and KKF supervised the calcium imaging and hIPSC generation, respectively, and edited the manuscript. MW co‐designed and coordinated the study, performed immunohistochemical stainings and edited the manuscript. All authors read and approved the final manuscript.

## PARTICIPANT CONSENT STATEMENT

Written informed consent for the use of brain tissue and clinical and neuropathological data for research purposes was obtained from all donors or their next of kin in accordance with the International Declaration of Helsinki and Europe´s Code of conduct for Brain Banking.

## Supporting information

Fig S1

Fig S2

Fig S3

## Data Availability

All data generated or analyzed during this study are included in this published article.
