# Supplementary figures and images for: Neuronal α‐amylase is important for neuronal activity and glycogenolysis and reduces in presence of amyloid beta pathology
Source: Aging Cell. 2021 Jul 14;20(8):e13433. doi: 10.1111/acel.13433 (PMC8373367; doi:10.1111/acel.13433)

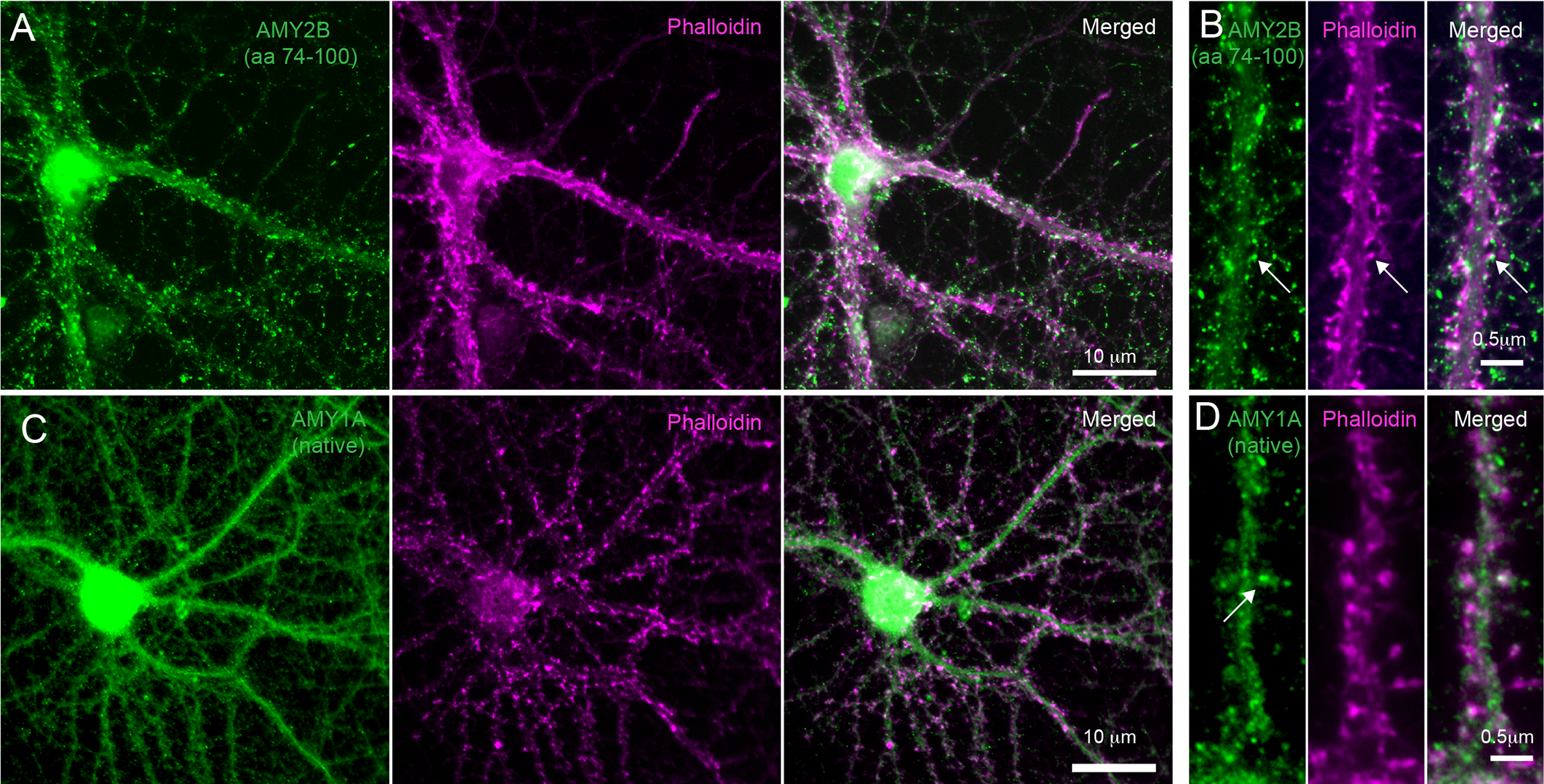

Supplement: Supplementary file 1 — Fig S1 [file ACEL-20-e13433-s003.tif]

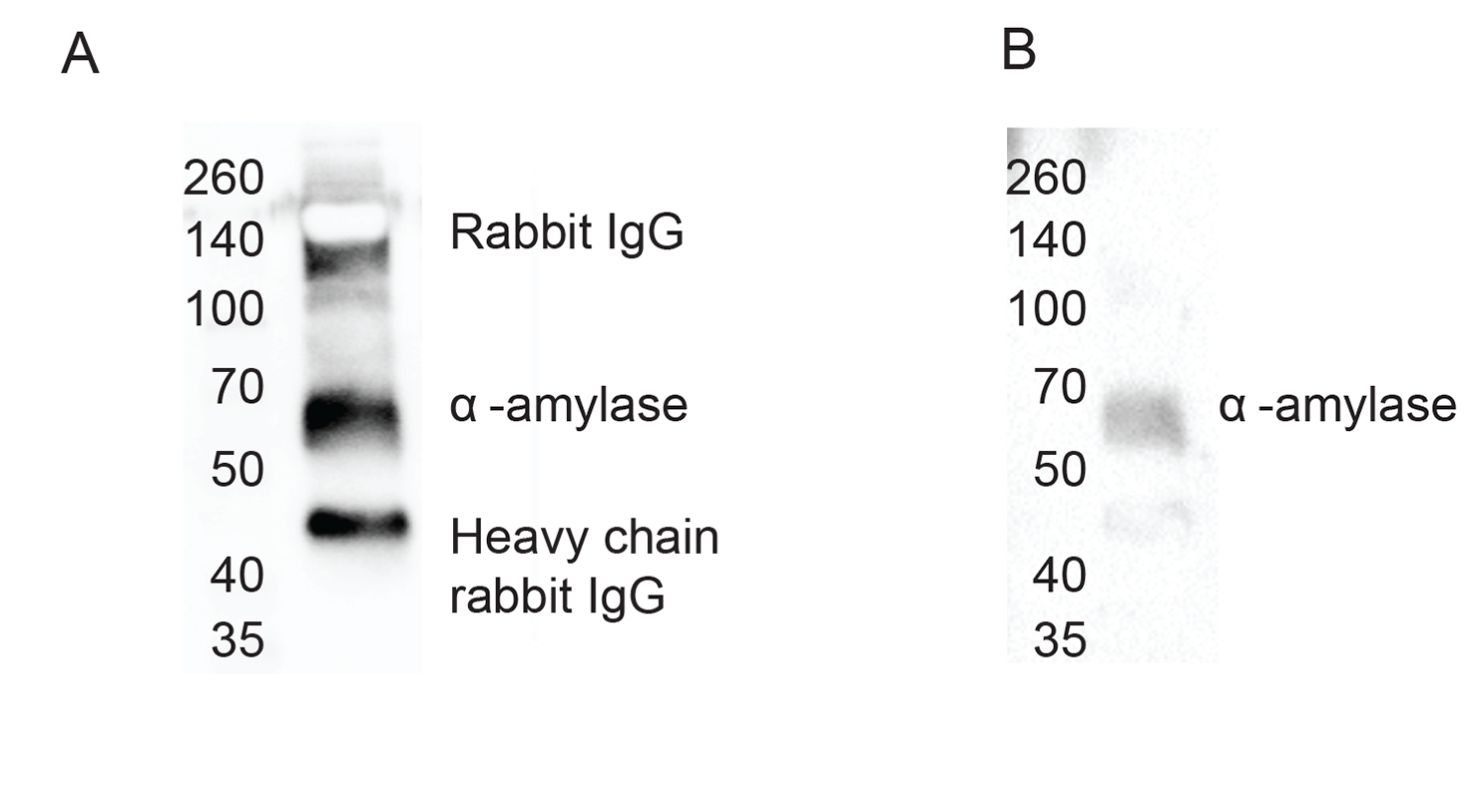

Supplement: Supplementary file 2 — Fig S2 [file ACEL-20-e13433-s001.tif]

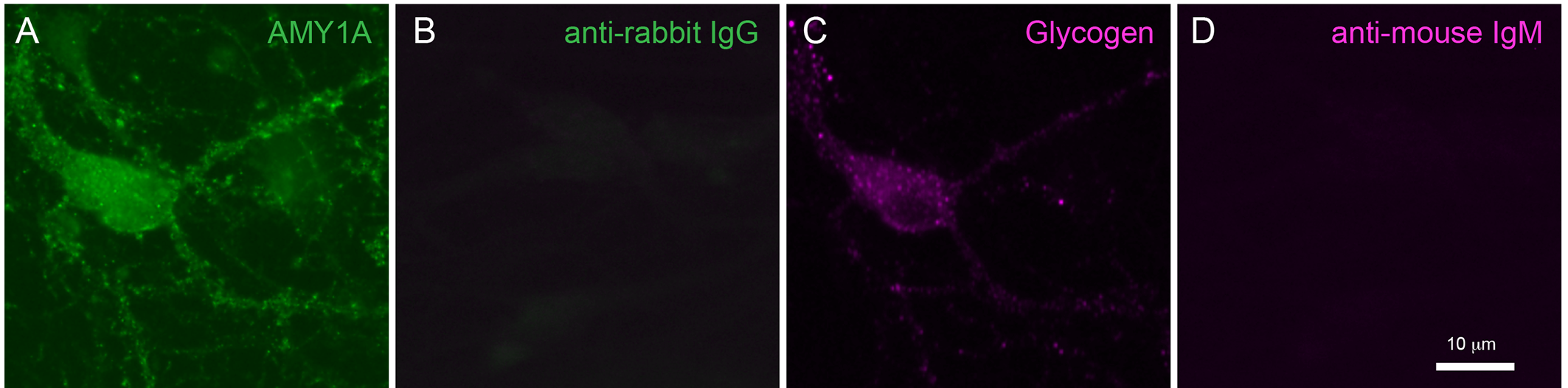

Supplement: Supplementary file 3 — Fig S3 [file ACEL-20-e13433-s002.tif]
